# Porf-2 = Arhgap39 = Vilse: A Pivotal Role in Neurodevelopment, Learning and Memory

**DOI:** 10.1523/ENEURO.0082-18.2018

**Published:** 2018-10-15

**Authors:** Felicia V. Nowak

**Affiliations:** Department of Biomedical Sciences, Diabetes Institute and Program in Molecular and Cellular Biology, Ohio University, Athens, OH 45701

**Keywords:** Apoptosis, Arhgap39, dendritic spines, learning and memory, Porf-2, RhoGAP

## Abstract

Small GTP-converting enzymes, GTPases, are essential for the efficient completion of many physiological and developmental processes. They are regulated by GTPase activating proteins (GAPs) and guanine nucleotide exchange factors (GEFs). *Arhgap39*, also known as preoptic regulatory factor-2 (Porf-2) or Vilse, a member of the Rho GAP group, was first identified in 1990 in the rat CNS. It has since been shown to regulate apoptosis, cell migration, neurogenesis, and cerebral and hippocampal dendritic spine morphology. It plays a pivotal role in neurodevelopment and learning and memory. Homologous or orthologous genes are found in more than 280 vertebrate and invertebrate species, suggesting preservation through evolution. Not surprisingly, loss of the *Arhgap39/Porf-2* gene in mice manifests as an embryonic lethal condition. Although *Arhgap39/Porf-2* is highly expressed in the brain, it is also widely distributed throughout the body, with potential additional roles in oncogenesis and morphogenesis. This review summarizes, for the first time, the known information about this gene under its various names, in addition to considering its transcripts and proteins. The majority of findings described have been made in rats, mice, humans, and fruit flies. This work surveys the known functions, functional mediators, variables modifying expression and upstream regulators of expression, and potential physiological and pathological roles of *Arhgap39/Porf-2* in health and disease.

## Significance Statement

This review comprehensively includes what is currently known about *Arhgap39/Porf-2* under its multiple names. Arhgap39 is a critically required molecule for neurodevelopment, learning, and memory that is expressed throughout the lifespan. It also has definitive roles in stem cell fate and cell migration, apoptosis, and proliferation, in both neural and nonneural sites. Homologous or orthologous genes have been conserved for millennia through evolution with a wide phylogenetic distribution from invertebrates to mammals. Its expression is exquisitely regulated by age, sex, location, and hormones. The consequences of dysregulation of *Arhgap39* are currently under investigation.

## Introduction

### GTP-converting enzymes and their modifying proteins

**Figure 1.**
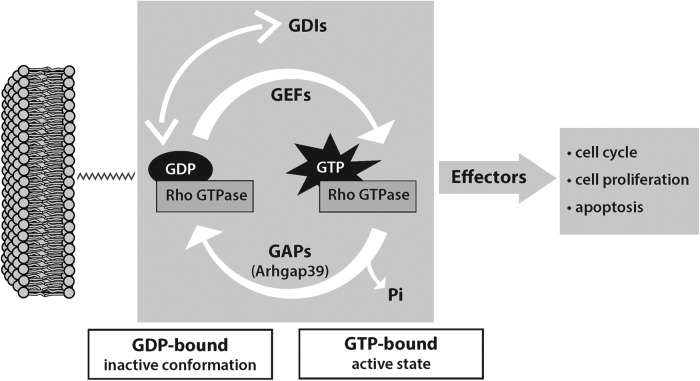
Schematic of Rac/Rho functional conversion, activation, and inactivation. GAP, GTPase activating protein; GDI, guanine nucleotide dissociation inhibitor; GEF, guanine nucleotide exchange factor; GTPase, guanine nucleotide triphosphate hydrolase.

Small GTP-converting enzymes, GTPases, ranging in size from 20 to 40 KDa, are essential for the efficient completion of many physiologic and developmental processes (Peck et al., 2002; Moon and Zheng, 2003; Jiang and Ramachandran, 2006; Tcherkezian and Lamarche-Vane, 2007; De Filippis et al., 2014; Ghosh et al., 2017). The GTPases switch between an active GTP-bound state and an inactive GDP-bound form as illustrated in Fig. 1. These switches are mediated by GTPase activating (or accelerating) proteins (GAPs) and guanine nucleotide exchange factors (GEFs). GEFs promote the binding of GTP, thus favoring the active state, while GAPs enhance the intrinsic activity of the GTPase, leading to GTP hydrolysis and returning the GTPase to an inactive state. Thus, GAPs can also be considered co-effectors with their GTPase partners. The inactive state is further promoted by guanine nucleotide dissociation inhibitors (GDIs), which sequester and stabilize the GDP-associated form.

There are five GTPase subfamilies: Ras (rat sarcoma), Rho (Ras homolog), Rab (rat brain), Arf/Sar (ADP-ribosylation factor/secretion-associated Ras-related protein), and Ran (Ras-related nuclear) in eukaryotes. These five subfamilies have been extensively studied in yeast, *Arabidopsis*, rice, *Drosophila melanogaster*, and selected vertebrates, especially rodents and humans (Jiang and Ramachandran, 2006; De Filippis et al., 2014; Yorimitsu et al., 2014). They regulate a diverse array of cellular functions, such as cytoskeletal reorganization, cell motility and polarity, vesicular trafficking, and cell fate and differentiation. Each GTPase subfamily has an associated GAP subfamily. Several groups of Rho GTPases exist, including Rho, Rac, Cdc42, and Rnd. An individual RhoGAP can interact with several Rho GTPases, depending on the cellular and developmental context.

### A brief history of Arhgap39/Porf-2 discovery

The first paper on *Arhgap39/Porf-2* reported the partial sequence and tissue distribution of the messenger RNA in the rat (Nowak, 1990). Subsequently, the investigation of the physiology and regulation of *Arhgap39/Porf-2* in the rat led to the publication of 11 peer-reviewed manuscripts, including a summary book chapter on the discovery and characterization of Porf-2 (Nowak, 2014). It was found that Arhgap39/Porf-2 proteins are encoded by multiple RNA transcripts with tissue-specific expression. *Arhgap39/Porf-2* is highly expressed in mammalian brain, including hypothalamus (regulation of metabolism and reproduction) and hippocampus (essential for learning and memory; Nowak, 1990; Hu and Nowak, 1994). The pattern of hypothalamic expression in the preoptic area-anterior hypothalamus (POA-AH) during a critical period of prenatal development is sex specific, with levels peaking earlier in males [embryonic day 18 (E18)–E19] than in females [postnatal day 0 (P0); Nowak and Gore, 1999]. It was found that the hormones, estradiol (E2) and progesterone (P4), regulate its expression in the female rat brain (Nowak et al., 1999). In males, gonadotropic pituitary factors decrease Arhgap39/Porf-2 mRNA in the POA and in the testes (Nowak et al., 1997) and testicular factors decrease Arhgap39/Porf-2 mRNA in the POA, but not in the medial basal hypothalamus (MBH) or cerebral cortex. Through this work it was also learned that *Arhgap39/Porf-2* is highly expressed in a subset of immature germ cells in testes (Nowak et al., 1997). In sum, these results suggest that precise regulation of *Arhgap39/Porf-2* expression has an impact on both CNS development and reproductive health. Finally it was discovered, using Southern blot analysis, that Arhgap39/Porf-2-like genes were present in nine widely ranging groups of vertebrates including primates, ungulates, rodents, birds, and fish (Schmerr et al., 2002), but the functions of this gene remained elusive. For more detail, see Variables modifying expression and upstream regulators.

### Arhgap39/Porf-2—from EST to ORF

Arhgap39/Porf-2, also known as Vilse, KIAA1688, D15Wsu169e, CrossGAP, and Rho GTPase activating protein39, is a RhoGAP protein. Its ortholog is known as *Arhgap93B* in *Drosophila*. Evidence that this gene contains a functional open reading frame (ORF) emerged slowly, initially based on a partial sequence (Nowak, 1990) from a rat brain cDNA library. Its existence was hinted at as a member of several expressed sequence tag (EST) collections (Ko et al., 1998; Miki et al., 2001; Okazaki et al., 2002), including pre-implantation mouse embryo (Ko et al., 2000), and orphan gene libraries (Bonaldo et al., 1996; Carninci et al., 2000). *Arhgap39/Porf-2* has been identified in more than 20 large-scale comprehensive enumerations of genes expressed as RNA transcripts and proteins. The complete mouse coding sequence was predicted in 2001 (Kawai et al., 2001) and verified by Okazaki et al. (2004). The Arhgap39/Porf-2 story has emerged slowly from a series of studies that demonstrate regional, temporal, and physiologic variations in expression, tissue, and location, some of which likely reflect species-specific functional roles for this gene. These will be discussed in more detail below.

## Gene, RNA, and Protein (Table 1)

### Phylogenetic distribution of Arhgap39/Porf-2

In 2002, the first phylogenetic distribution analysis was reported for *Arhgap39/Porf-2* (Schmerr et al., 2002). Southern blot analysis using a rat probe revealed homologs in human, mouse, pig, sheep, cow, chicken (two bands), and zebrafish. It was shown that, in the rat, *Arhgap39/Porf-2* is a single-copy gene (Nowak et al., 1997), and this has been confirmed in human and mouse with the sequencing of their complete genomes. In *Drosophila*, there is a single ortholog. However, the possibility remains that duplications have occurred in other species, including chickens.

**Table 1.** Arhgap39/Porf-2 gene structure, RNA transcripts, and proteins

Gene structure
The human gene is more than 171,000 base pairs in length and contains 16 exons
The mouse homolog is 94,000 base pairs long and has 13 exons
The rat homolog is 92,000 base pairs long and has 13 exons
Homologues/orthologs identified in >190 vertebrate species
>100 additional orthologs identified in nonvertebrate animal species
Chromosome locations are on 15 in mouse, 8 in human, and 7 in rat
mRNA transcripts
3 verified and 5 predicted in human (*H. sapiens*)
2 verified and 1 predicted in mouse (*Mus musculus*)
1 verified and 5 predicted in rat (*R. norvegicus*)
1 verified in *Drosophila*
Proteins, domains, and motifs
Proteins range in size from 16.5 to 133 kDa
Domains include WW, MyTH4, and RhoGAP
Additional predicted motifs include serine and threonine phosphorylation, SH3, and SH2

In 2002, the Mammalian Gene Collection Program team identified murine *Arhgap39/Porf-2* as a candidate full-ORF clone (Strausberg et al., 2002). Subsequently, in 2004, the Mammalian Genome Project reported the full-ORF cDNA sequences in mouse and human (Gerhard et al., 2004). In 2011, the mouse gene location was determined by the Jackson Laboratory (Bar Harbor, ME) to be on chromosome 15 (Diez-Roux et al., 2011). The gene is positioned on chromosome 7 in rats and on chromosome 8 in humans.

To date, 180 orthologs, identified by gene and exome sequencing, have been reported in a variety of vertebrate species, from platypuses (https://www.ncbi.nlm.nih.gov/gene/103168041) to equids (Hestand et al., 2015) and penguins (https://www.ncbi.nlm.nih.gov/gene/103901269). Moreover, the homologous mouse and human genes are located in a large conserved syntenic region corresponding to human chromosome 8 and mouse chromosome 15, supporting derivation from a common ancestor that existed at minimum 75 million years ago (Mouse Genome Sequencing Consortium et al., 2002). A related gene (*Arhgap93B*) in *Drosophila* and other invertebrates has now been identified in >100 additional species. As the common ancestor of vertebrates and insects existed at minimum ∼530 million years ago, this raises the possibility that *Arhgap39/Porf-2* is even older, but this idea remains to be rigorously evaluated.

### DNA and RNA structure and modifications

The mouse *Arhgap39/Porf-2* gene is ∼94,000 base pairs in length and contains 13 exons. The *Rattus norvegicus* gene is slightly shorter at ∼92,000 bp, also with 13 exons. The *Homo sapiens* gene is ∼171,000 bp with 16 exons. The arrangement and spacing of the 12 3′-most exons are similar in these three species; the increased length of the human gene appears to reflect extensions of the introns upstream of the fourth rat/mouse exon. Whereas the *Drosophila* counterpart is only 14,785 bp, it has 14 exons and 3 domains that are highly conserved within the vertebrate genes noted above; these are WW, myosin tail homology 4 (MyTH4), and GAP domains described below.

**Figure 2.**
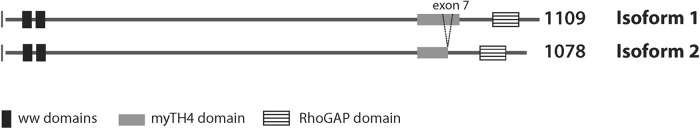
Sequences of two isoforms of Arhgap39/Porf-2 protein in mouse. The protein sequence numbers correspond to those in the NCBI database. Adapted from https://www.ncbi.nlm.nih.gov/gene/223666 (mouse). The functional domains include two WW, and one each of MyTH4 and GAP. Protein isoform 2 differs from isoform 1 in the deletion of 31 amino acids, encoded by exon 7, from the MyTH4 domain.

Through *in silico* and Northern blot analyses, as well as sequencing of full-length cDNAs in human, rat, and mouse, it has been clearly demonstrated that the *Arhgap39/Porf-2* gene can give rise to multiple RNA transcripts (Nowak, 1990, 1997; Rietveld et al., 2014). Although it was briefly reported that there was a second related gene in the mouse (Lundström et al., 2004), it was subsequently shown to represent an alternative splice form of Arhgap39/Porf-2 mRNA. However, there is evidence that two of the major transcripts give rise to slightly different proteins, owing to alternative splicing of the in-frame exon 7 (Fig. 2). The mouse protein isoforms 1 and 2 differ by elimination of exon 7 in isoform 2, which results in a 31-amino-acid deletion in the MyTH4 domain. Human isoforms 1 and 2 are similar to mouse isoform 2 and the single verified rat isoform in that they all encode a protein that lacks exon 7. Human isoforms 1 and 2 differ in their 5′ untranslated regions. In addition to the verified transcripts, there are a number of predicted transcripts. To a large extent, the resulting protein or other products of these transcripts have not been characterized. Alternative splicing occurs in 41% of mouse transcriptome and, in transcripts which contain a coding sequence, 79% of splice variations alter the protein products, and potentially, their subcellular localization and function (Okazaki et al., 2002). It remains to be seen whether there are different forms of Arhgap39/Porf-2 protein in different subcellular compartments or with alternative posttranslational modifications that allow for it to have several separate functions.

### Protein domains and modifications

The RhoGAP family members are notable for having multiple functional domains in addition to the GAP domain, including many that have been implicated in protein–protein interactions. This organization may facilitate interactions with more than one signaling pathway (Moon and Zheng, 2003; Tcherkezian and Lamarche-Vane, 2007). In the mouse, as well as in *Drosophila*, Arhgap39/Porf-2 protein has been shown to have three potentially functional domain regions, as shown in Fig. 2.

### Two WW domains

The first hypothesized functional region is represented by a pair of WW domains at the N terminus of the predicted *Drosophila* protein (Lundström et al., 2004) and mouse isoform 1 (Lim et al., 2014). WW domains are ∼35–45 amino acids in length and are characterized by the presence of hydrophobic amino acids with cyclic side chains, including tryptophan, tyrosine, phenylalanine, and proline, but do not usually otherwise exhibit significant homology between different genes. However, the two WW domains of human Arhgap39/Porf-2 exhibit 84% and 77% sequence similarity, respectively, with the related drosophila *Arhgap93B* (Lundström et al., 2004). WW domains recognize and interact with proline-rich segments of other proteins to facilitate protein–protein interactions (Otte et al., 2003).

### MyTH4 domain

The second region is a MyTH4 domain, an ∼150-amino-acid module. MyTH4 domains have been identified as a conserved sequence in the tail domains of several different unconventional myosins and a plant kinesin-like microtubule protein. There is a 44% protein sequence similarity between the *H. sapiens* Arhgap39/Porf-2 and *Drosophila* Arhgap93B MyTH4 domains (Lundström et al., 2004). Although the function of MyTH4 domains is not yet fully understood, there is evidence that the MyTH4 domain of Myosin-X (Myo10) binds to microtubules (Kerber and Cheney, 2011). The MyTH4 domain has also been found in several nonmotor proteins. Interestingly, the results of Huang et al. (2002) suggest a possible role for one of these (MAX-1) in netrin-induced axon repulsion in *Caenorhabditis elegans* by modulating the UNC-5 receptor signaling pathway. MAX-1 homologues are also found in *Drosophila*, mouse, and human (Kikuno et al., 2000; Huang et al., 2002). An emerging theme is that many, and perhaps most, guidance cues are bifunctional. Arhgap39/Porf-2 and MAX-1 are both MyTH4 domain–containing proteins that display this attraction-repulsion dichotomy.

### RhoGAP domain

The third functional region is a RhoGAP domain close to the C terminus. RhoGAP domains are ∼140 amino acids, sharing 20%–40% amino acid identity (Lamarche and Hall, 1994). The actual GTPases with which they interact cannot be predicted by the sequence but must be experimentally determined. The human Arhgap39/Porf-2 GAP domain shares 86.5% homology with the rat, 88.8% with the mouse (personal calculation), and 61% with *Drosophila* (Lundström, Gallio, 2004). The rat also has transcripts that include the RhoGAP domain; however, it also has at least two variants, X4 and X5, that lack this domain. Instead, each contains a distinct C-terminal sequence.

### Additional motifs and modifications

In addition to the WW, MyTH4, and GAP domains, the predicted protein isoforms 1 and 2 have 19 potential motif sites, including 8 serine/threonine phosphorylation sites, 2 SH2, and a single SH3 motif (Obenauer et al., 2003). Serine or tyrosine phosphorylation of regulatory proteins including GAPs is one mechanism by which activation and subcellular location can be controlled. One example is CdGAP, a RhoGAP whose activity is regulated through phosphorylation by ERK (Tcherkezian et al., 2005). Another example is the phosphorylation of the RacGAP protein, FilGAP, at Ser-402 (Morishita et al., 2015). Phosphorylation increases its RacGAP activity *in vivo* and promotes diffuse distribution in the cytoplasm, which stimulates cell spreading on fibronectin. The total number and active sites of phosphorylation in the Arhgap39/Porf-2 protein have not been determined. One of the predicted phosphorylation sites in Arhgap39/Porf-2 at serine 169, within a casein kinase I motif, was confirmed by a screen of mouse synaptosomal proteins isolated from 2–5-month cortex (Munton et al., 2007). In addition, phospho-serine 589 was identified in a large screening of the developing mouse brain (E16.5) for phosphorylated peptides by Ballif et al. (2004); interestingly, this was not one of the phosphorylation sites previously predicted.

SH2 and SH3 are homology domains found in a family of oncogenic nonreceptor tyrosine kinases. An SH2 domain, which was first identified in the oncoproteins Src and Fujiyami poultry sarcoma, is ∼100 amino acid residues long. It functions as a regulatory module of intracellular signaling cascades by interacting with high affinity to phosphotyrosine-containing target peptides in a sequence-specific and strictly phosphorylation-dependent manner.

SH3 domains are small protein domains of ∼60 amino acid residues. SH3 domains are found in proteins of signaling pathways regulating the cytoskeleton, the Ras protein, the Src kinase, and many others. SH3 domains interact with other proteins and mediate assembly of specific protein complexes, typically via binding to proline-rich peptides in their respective binding partner.

The presence of these motifs that accelerate protein localization and interactions in Arhgap39/Porf-2 are consistent with its role as a RhoGAP that is involved in several complex signaling pathways.

## Tissue distribution of Arhgap39/PORF-2 RNAs and proteins (Table 2)

The first partial Arhgap39 (Porf-2) sequence was reported in a cDNA library constructed with RNA isolated from adult rat hypothalamus (Nowak, 1990). This group detected multiple poly A–enriched RNA transcripts expressed widely in the adult rat, including cerebral cortex, hippocampus, hypothalamus, cerebellum, amygdala, anterior pituitary, adrenal, testis, liver, and kidney (Nowak, 1990, 1997, 2014). ESTs and alternative splicing sequence enriched tags (ASSETs; Watahiki et al., 2004) were also found in E7.5 mouse embryo placental growth cone (Ko et al., 1998) and E17.5 mouse skin and testes (Miki et al., 2001). In 2001, human KIAA1688 cDNA was sequenced, and human tissue distribution of Arhgap39/Porf-2 RNA was quantified by PCR (Kikuno et al., 2004). Although RNA levels were highest in brain and spinal cord, it was also found in testis, kidney, liver, pancreas, lung, and other tissues at lower levels.

**Table 2.** Tissue and cell type distribution of Arhgap39/Porf-2 expression in human, mouse, rat, and fruit fly

Type of tissue or cell	Location (species)	Method of detection
Brain	Hippocampus (R, M, H)	N, NPA, RNAi, IHC
	Hypothalamus (R)	N, NPA, ISH
	Cerebral cortex (R, M)	N, NPA, IHC; RNA Seq (Yue et al., 2014)
	Cerebellum (R, M)	N, RNA Seq (Yue et al., 2014)
	Amygdala (R)	ISH
	Ventral neuropil (D)	ISH (Lundström et al., 2004)
Peripheral nervous system	Tracheal ganglia (D)	RNAi (Lundström et al., 2004)
Endocrine organs	Testes (R, H, M)	N, ISH, EST
	Anterior pituitary (R)	N
	Adrenal (H, M, R)	N, RNA Seq (Yu et al., 2014; Yue et al., 2014)
	Placenta (H, R, M)	N, EST (Ko et al., 2000); RNA Seq (Yu et al., 2014; Yue et al., 2014)
	Ovary (M, H)	RNA Seq (Yue et al., 2014; Fagerberg et al., 2014)
	Adipose tissue (M, H)	RNA Seq (Yue et al., 2014; Fagerberg et al., 2014)
	Mammary gland (M)	RNA Seq (Yue et al., 2014)
	Thyroid (H)	RNA Seq (Fagerberg et al., 2014)
	Pancreas (H)	RNA Seq (Fagerberg et al., 2014)
Other organs	Prostate (H, R)	N, RNA Seq
	Liver (R, H, M)	N, PCR, WB; RNA Seq (Yue et al., 2014)
	Skin (M, H)	EST (Miki et al., 2001); RNA Seq (Fagerberg et al., 2014)
	Uterus (R, H)	RNA Seq (Yu et al., 2014; Fagerberg et al., 2014)
	Lung (R, H)	RNA Seq (Yu et al., 2014; Fagerberg et al., 2014)
	Spleen (R, H)	RNA Seq (Yu et al., 2014); RNA Seq (Fagerberg et al., 2014)
	Heart (R, M)	RNA Seq (Yu et al., 2014; Yue et al., 2014)
	Thymus (R, M)	RNA Seq (Yu et al., 2014; Yue et al., 2014)
	Skeletal Muscle (R, M)	RNA Seq (Yu et al., 2014; Yue et al., 2014)
	GI tract (M, H)	RNA Seq (Yue et al., 2014; Fagerberg et al., 2014)
	Bladder (M)	RNA Seq (Yue et al., 2014)
	Kidney (R, H)	PCR; RNA Seq (Fagerberg et al., 2014)
Cells	FRTL-5 (R thyroid-like)	WB, PCR
	Pancreatic beta cells (H)	PCR (Wang, 2011)
	cos7 cells (nhP kidney)	PAGE
	C17.2 (M cerebellar stem)	PCR, WB
	GT-1 (M hypothalamic neuron)	WB
	Vascular endothelial (H)	IP (Kaur et al., 2008)
	Melanocytes (H)	ASSET (Watahiki et al., 2004)

D, *Drosophila*; H, human; M, mouse; nhP, nonhuman primate; R, rat; ASSET, alternative splicing sequence enriched tag; ISH, in situ hybridization; IP, immunoprecipitation; IHC, immunohistochemistry; N, Northern blot; NPA, nuclease protection assay; WB, Western blot. Unless otherwise noted, determinations were made in the authors lab.

RNA transcripts have also been detected in human hypothalamus, adrenal, prostate, and placenta (Nowak, 2014) and in several cultured cell types, including human melanocytes (Watahiki et al., 2004), human vascular endothelial cells (Kaur et al., 2008), mouse cerebellar stem cells (C17.2; Ma and Nowak, 2011), Fisher rat thyroid-like (FRTL-5) cells, mouse hypothalamic neurons (GT-1; Nowak, 2014), and human pancreatic beta cells (Wang J., 2012, personal communication). Recently, several extensive studies of gene expression in human (Fagerberg et al., 2014), mouse (Yue et al., 2014), and rat (Yu et al., 2014) have expanded the molecular distribution of Arhgap39/Porf-2 RNA to include intestine, spleen, heart, thymus, uterus, lung, stomach, ovary, urinary bladder, mammary gland, prostate, salivary gland, thyroid gland, and adipose tissue.

Ballif et al. (2004) identified an Arhgap39/Porf-2 phosphopeptide in a large proteomic analysis of E16.5 mouse brain. Diez-Roux et al. (2011) found moderate protein expression of Arhgap39/Porf-2 (D15Wsu169e) in E14.5 mouse embryo brain and spinal cord and peripheral nervous system, and low expression in multiple other peripheral locations, including renal, hematopoietic, gastrointestinal, and smooth and striated muscle. Arhgap39/Porf-2 protein expression was also reported in mouse cerebellar stem cells (Ma and Nowak, 2011). Recently, several Arhgap39/Porf-2 proteins were detected in human male liver (unpublished observations, Liu, Zhang, Guo, and Nowak).

In the brain, Arhgap39/Porf-2 RNA was first localized to neurons (Nowak et al., 1999) in 60-d-old female rat hypothalamus by *in situ* hybridization analysis. It was later detected as a phosphoprotein by liquid chromatography-tandem mass spectrometry in synaptic terminals isolated from 2–5-month C57Bl/6 mouse cortex (Munton et al., 2007). Arhgap39/Porf-2 is also detected in neurospheres derived from mouse hippocampus (Huang et al., 2016).

## Functional roles and downstream effectors (Table 3)

Arhgap39/Porf-2 has been shown to function through regulation of two Rho GTPases, Rac1 and Cdc42. Several investigators have shown that Arhgap39/Porf-2 is a regulator of Rho GTPases and as such plays a role in endothelial cell migration (Kaur et al., 2008), ganglion and axon tracking (Lundström et al., 2004; Hu et al., 2005), neural stem cell fate (Ma and Nowak, 2011; Huang et al., 2016), and dendritic spine formation (Lim et al., 2014; Lee et al., 2017). These studies further link Arhgap39/Porf-2 to angiogenesis, tracheal innervation, and CNS development.

**Table 3.** Functions and mediators of Arhgap39/Porf-2

Functions	Mediators of action
1. Mediates axonal guidance and midline repulsion in developing *Drosophila*.	1. Robo, RacGTP, Slit (Lundström et al., 2004; Hu et al., 2005)
2. Regulates filopodia formation and directional migration of human vascular endothelial cells.	2. Robo1 and Robo4, Cdc42, IRSp53, MENA, actin nucleation (Kaur et al., 2008)
3. Delays cell cycle and decreases proliferation of mouse neural stem cell line, C17.2.	3. p21, decreased progression from G1 to S (Ma and Nowak, 2011)
4. Promotes apoptosis in C17.2 cells.	4. BAX, p53 (Ma and Nowak, 2011)
5. Regulates dendritic spine morphology in embryonic rat hippocampal neurons.	5. CNK2, Rac1 (Lim et al., 2014)
6. Decreases neurogenesis in mouse hippocampus.	6. Rac1, β-catenin (Huang et al., 2016)
7. Plays a role in learning and memory.	7. Hippocampal synaptic signaling (Lee et al., 2017)

### Axon directional migration

Lundström et al. (2004) were the first to show a functional role for *Arhgap93B*, the *Drosophila* ortholog of *Arhgap39/Porf-2*. They demonstrated that the WW domains of Vilse, as they termed it, bind to the CC2 domain of the Slit receptor, Roundabout (Robo), and inactivate RacGTP to mediate Slit-directed midline repulsion of CNS axons in tracheal cells. They further demonstrated that both *Drosophila* Arhgap93B and human Arhgap39/Porf-2 GAP domains effectively stimulated GTP hydrolysis of Rac1. Soon thereafter, Hu et al. (2005) identified Vilse, which they called CrossGAP, as a regulator of midline axonal repulsive guidance in *Drosophila*. They confirmed the direct interaction between Vilse and Robo and the inactivation of Rac1 by Vilse. Interestingly, either too little or too much Vilse resulted in defective Robo-mediated, Slit-directed axon repulsion at the midline.

### Endothelial cell migration

In human vascular endothelial cells, Arhgap39/Porf-2/Vilse is sequestered by Robo1 and Robo4, resulting in an increase in activated Cdc42. Cdc42-GTP then activates insulin receptor protein 53 (IRSp53) by binding to its CRIB domain. This exposes an SH3 domain that binds to Mena, an IRSp53 effector and mediates actin nucleation, resulting in filopodia formation and endothelial cell directional migration (Kaur et al., 2008).

### Neural stem cell fate

In 2011, the roles of Arhgap39/Porf-2 in the mouse neural stem cell (NSC) line, C17.2, were discovered, including its negative effects on cell cycle and proliferation and potentiating effects on apoptosis. The key cell signaling components involved in mediating these effects were identified (Ma and Nowak, 2011). These include increased levels of p21 leading to cell cycle arrest and decreased cell proliferation, and enhanced drug-induced apoptosis through both p53-dependent and -independent pathways by upregulating Bcl-2 associated X protein (BAX). These findings are consistent with those of others who have shown that BAX and p53 are regulators of programmed cell death in mouse cerebellum (Geng et al., 2010) as well as with the finding that upregulation of BAX and p53 can suppress the growth of tumors, including gliomas (Zhen et al., 2017).

This work also demonstrated that Arhgap39/Porf-2 had no effect on NSC differentiation induced by serum starvation (Ma and Nowak, 2011). Although this study did not identify the GTPase substrate involved, Ras, Rho, and Cdc42 have all been shown to regulate cell cycle entry and cell proliferation (Olson et al., 1995; Olson et al., 1998; Welsh et al., 2001) and to determine the fate of NSCs by influencing the balance of stem cell maintenance with proliferation and apoptosis (Joseph and Hermanson, 2010). Other RhoGAPs including DLC2 (Ching et al., 2003) and tGAP1 (Modarressi et al., 2004) have been shown to decrease cell proliferation by slowing down the cell cycle. tGAP1 also induces apoptosis when overexpressed in somatic cells.

Huang et al. (2016) showed that the effects of Arhgap39/Porf-2 on cell proliferation of NSCs were mediated through its GAP domain. They extended previous *in vitro* findings in C17 cells that Arhgap39/Porf-2 is anti-proliferative (Ma and Nowak 2011) and demonstrated that Arhgap39/Porf-2 decreases proliferation in neurospheres from newborn mouse hippocampus as well as in hippocampal sections from 6-week-old mice (Huang et al., 2016). Transfection with a full-length *Arhgap39/Porf-2* construct, but not one lacking the GAP domain, resulted in decreased levels of intranuclear β-catenin. The authors postulated that the observed effects on cell proliferation are mediated through the GAP domain, causing decreased translocation of β-catenin into the nucleus.

### Dendritic spine morphology

Lim et al. (2014) demonstrated that Arhgap39/Porf-2 is required for normal dendritic spine formation in the rat dentate gyrus, a portion of the hippocampal formation, and in mouse neuroblastoma × rat glioma hybrid cells. Interaction of a proline motif in the neuron scaffold protein connector enhancer of kinase suppressor of ras2 (CNK2) with the WW domain of Vilse regulates Arhgap39/Porf-2 localization. This critical interaction maintains the RacGDP/GTP balance required for dendritic spine formation. It is of note that CNK2 has been implicated in X-linked cognitive impairment in a human subject (Houge et al., 2012). Additionally, constitutive lack of the RhoGAP, oligophrenin 1, in mice results in decreased density and increased length of dendritic spines in the amygdala (Khelfaoui et al., 2014) and failure of hippocampal CA1 dendritic spines to mature during development (Khelfaoui et al., 2007). These mice also display deficits in learning and memory as measured with Y-maze, O-maze, Morris water maze, and conditioned fear extinction, as well as altered hippocampal LTP. Deficits in mice of additional Rho GTPase regulators, including SRGAP3, BCR and ABR, ARG, ABL, Integrin 3α, KALRN, and ARGEF6, have also been associated with cognitive and anxiety-related behavioral alterations, such as decreases in novel object recognition and Y-maze alternation and social interactions, altered synaptic plasticity, and abnormal dendritic spine morphology (De Filippis et al., 2014). Finally, exome sequencing of individuals from a family with late-onset Parkinson’s disease identified a mutation (p.Arg667Gln) in *Arhgap39/Porf-2* as a rare variant possibly associated with the disease (Schulte et al., 2014), indicating that dysregulation of this gene may also play a role in neurodegeneration.

### Learning and memory

In 2014, *Arhgap39/Porf-2* was among the genes identified to be associated with cognitive performance in humans (Rietveld et al., 2014). This may reflect variation in expression of mRNA variants 1 and 2, an interesting observation, since they both encode the same protein isoform, but differ in the 5′ UTR. Quite recently, Lee et al. (2017) described the effects in hippocampus of a widespread *Arhgap39/Porf-2* knockout in mouse forebrain under the direction of the CamKII promoter. These mice show behavioral deficits in learning and memory as measured by Morris water maze and Y-maze performance. They also exhibit abnormal hippocampal signaling and dendritic spine morphology. Not surprisingly, given its high level of expression in placental growth cone and developing nervous system, a global constitutive knockout resulted in embryonic lethality, with incomplete embryo development.

Taken together, these findings indicate that Arhgap39/Porf-2 is an integral part of the signaling machinery that regulates hippocampal structure and function and more broadly neuronal function, through its effects on neuronal cell proliferation and apoptosis, its regulation of axonal migration, and its role in dendritic spine morphology, synaptic plasticity, and cognitive performance.

### Oncogenesis

More recently, several bioinformatics analyses have uncovered associations of *Arhgap39/Porf-2* mutations or variations in copy number or expression level with several types of cancer (https://cancer.sanger.ac.uk/cosmic/gene/analysis?ln=ARHGAP39, http://useast.ensembl.org/Homo_sapiens/Gene/Variation_Gene/Table?db=core;g=ENSG00000147799;r=8:144529179-144605816). These include tumors of the CNS, skin, prostate, and gastrointestinal tract; one conserved variant has been shown to correlate with prostate tumors and sarcomas in a family lineage (Jones, 2017). Given its roles in apoptosis and proliferation and interactions with p53 and BAX, a mutation that decreased expression of *Arhgap39/Porf-2* could result in increased cell proliferation, leading to tumorigenesis.

## Variables modifying expression and upstream regulators of Arhgap39/PORF-2 (Table 4)

Whereas it is important to know the downstream effects of Arhgap39/Porf-2 action, it is also critical to understand the physiologic factors that regulate *Arhgap39/Porf-2* expression. It has been known for some time that Arhgap39/Porf-2 mRNA in the rat brain is localized mainly to neurons (Nowak et al., 1999) and that in the rat, age, sex differences, brain region, gonadal steroids, and developmental stage regulate/influence the expression of Arhgap39/Porf-2 mRNAs in hippocampus, hypothalamus, and cerebral cortex (Hu and Nowak, 1994, 1995; Nowak, 1997; Nowak and Gore, 1999; Nowak et al., 1999).

**Table 4.** Variables modifying expression and upstream regulators of Arhgap39/Porf-2

Developmental stage (rat brain)
Age (rat brain and testes)
Sex (rat brain)
Brain region (rat)
Gonadal hormones, including estradiol and progesterone (rat brain)
Pituitary factors (rat brain and testes)
Epigenetic mechanisms such as miRNAs
Insulin and IGF-1 (FRTL-5 cells)
Type 2 diabetes (Zucker rat kidney)
Obesity (human liver)

### Developmental and age-related changes in CNS expression of Arhgap39/Porf-2

As measured by nuclease protection assays of cytoplasmic RNA, Arhgap39/Porf-2 RNA averages 40 μg/g total RNA from 15 d to 2 months of age in the male rat hippocampus. The levels increase more than 2-fold at 6 and 12 months, then decline with age. Why Arhgap39/Porf-2 is highest in the male rat hippocampus at middle age remains an unsolved question. Prenatal Arhgap39/Porf-2 RNA levels in the POA are high at E18–E19, then fall gradually until P15. This is followed by a plateau through 2 months of age when postnatal levels are highest (19.9 μg/g) followed by a progressive decrease with age (Hu and Nowak, 1994). Postnatal age-related changes in expression have also been identified in the MBH and cerebral cortex (Hu and Nowak, 1995), where Arhgap39/Porf-2 RNA is severalfold higher in both male and female rats at 15 d compared to 30 and 60 d, followed by a long plateau through 24 months of age. Arhgap39/Porf-2 RNA in the MBH also declines rapidly by 2-fold from E18–E19 to P15 in both sexes (Nowak and Gore, 1999). Its regionally distinctive, developmentally modified neuronal expression suggested early on that *Arhgap39/Porf-2* has a critical role in prenatal and postnatal CNS development and function.

### Sex differences in the developing CNS

There are also sex differences in Arhgap39/Porf-2 cytoplasmic RNA levels during development in the rat, being higher in male than in female hippocampus and MBH at P15 (Hu and Nowak, 1995). In the POA, late embryonic levels are higher in males at E18–E19, then decline rapidly, resulting in lower levels in males at P0 and P15 (Nowak and Gore, 1999). Thus there is a sex difference in timing for peak levels of Arhgap39/Porf-2 in the developing rat fetal POA, a brain region that is critical for normal feedback regulation of gonadotropin-releasing and -inhibiting hormones and reproductive function more generally.

### Gonadal hormones

In the adult rat, *Arhgap39/Porf-2* expression is responsive to extrinsic variations in circulating gonadal hormones in both females and males. In females ovariectomized at 44 days, treatment with E2 alone results in an increased level of Arhgap39/Porf-2 RNA in the POA and hippocampus compared with placebo-treated controls (Nowak et al., 1999). Replacement of only P4 upregulates Arhgap39/Porf-2 in POA and cerebral cortex. Treatment with E2 plus P4 also increases Arhgap39/Porf-2 in the hippocampus, but this combination decreases the RNA in POA and cortex. No changes are observed in the MBH. Thus the response of the female rat brain to gonadal steroids is highly region dependent. Based on Northern blot analysis of Arhgap39/Porf-2 mRNA in male rats subjected to hypophysectomy or castration, both pituitary and testicular factors directly or indirectly affect alternative splicing of Arhgap39/Porf-2 mRNA transcripts in the POA, MBH, and cerebral cortex (Nowak, 1997). At the present time, it is not known how this specifically impacts sex-related gene function or how *Arhgap39/Porf-2* mediates the effects of reproductive axis hormones in the brain.

### Pituitary factors

Expression of Arhgap39/Porf-2 RNA in rat testes is predominantly localized to spermatogonia and primary spermatocytes and is regulated by age and by pituitary gonadotrophic hormones (Nowak et al., 1997). Arhgap39/Porf-2 testicular expression declines with age in the rat (Nowak et al., 1997; Yu et al., 2014). At 60 d of age, Arhgap39/Porf-2 RNA has declined to 40% of the level at 15 d; there is a further 50% decline between 60 d and 6–12 months. By 24 months, the level is 8%–28% of that at 15 d. Hypophysectomy of young adult male rats results in a significant increase in testicular Arhgap39/Porf-2 to levels normally seen at 15 d. Although the developmental pattern of expression, in addition to the germ cell location and regulation by hormones of reproduction, suggests a function for Arhgap39/Porf-2 proteins in mammalian testes, that specific function is currently unknown. Another RhoGAP, tGAP1, which is mainly found in testes, has been shown to be expressed later, in round spermatids, where it slows down the cell cycle and may induce apoptosis (Modarressi et al., 2004).

### Epigenetic mechanisms

Recently, epigenetic mechanisms have been implicated in the regulation of *Arhgap39/Porf-2* expression (https://ccb-web.cs.uni-saarland.de/mirtargetlink/network.php?type=Target_Gene&qval=ARHGAP39). miRTargetLink currently lists 23 miRNAs with weaker experimental evidence that may target *Arhgap39/Porf-2*.

### Metabolism, insulin, and IGF-1

More recently, it was discovered that *Arhgap39/Porf-2* expression is also modified by two important regulators of metabolism and growth, insulin and IGF-1 (Wang, 2011). Both insulin and IGF-1 decrease *Arhgap39/Porf-2* expression in FRTL-5 cells in a dose- and time-dependent manner. That this downregulation is signaled through the PI-3-kinase/AKT and Raf kinase/MEK pathways was shown by use of specific chemical inhibitors, including wortmannin and Ly29402 for PI-3-K and Akt inhibitor-4, Raf kinase inhibitor, and high-dose wortmannin and PD98059 for MEK/Erk phosphorylation. Additionally, knockdown of IGF-1 receptor (IGF-1R) and possibly insulin receptor results in a partial reversal of the downregulation. An additional study of interest (Bentov et al., 2014) showed an association of IGF-1R deficiency in aged dermal fibroblasts with decreased Erk phosphorylation and decreased cell proliferation. Although *Arhgap39/Porf-2* is expressed in skin and is a potential mediator of this effect, neither it nor other potential downstream regulators were measured in this study.

It makes physiologic sense that stimulators of growth and metabolism would act counter to Arhgap39/Porf-2, a regulator that promotes apoptosis and inhibits cell proliferation. Recent findings have shown that neurogenesis is impaired and apoptosis increases in hippocampi of subjects with diabetes (Ho et al., 2013). A logical question that follows is, what happens to endogenous levels of Arhgap39/Porf-2 when insulin or IGF-1 is deficient as in type 1 diabetes or growth hormone deficiency, either congenital (Laron syndrome) or age-related (Gong et al., 2014), or if the response to insulin or IGF-1 is blocked, as seen in obesity with insulin resistance, type 2 diabetes, or aging (Bentov et al., 2014)?

### Insulin resistance and obesity

Two recent findings suggest a link between insulin resistance and *Arhgap39/Porf-2*. In a Zucker rat model of obesity-related diabetes, treatment with an antioxidant-fortified diet resulted in partial preservation of glomerular filtration rate in 20-wk-old diabetic females (Slyvka et al., 2009). This group also found decreased expression of Arhgap39/Porf-2 RNA in the kidney compared to diabetic females on a control diet. Obese humans and rodents also have been shown to have decreased hepatic expression of Arhgap39/Porf-2 and carcinoembryonic antigen related cell adhesion molecule 1 (Heinrich et al., 2017). Decreased carcinoembryonic antigen related cell adhesion molecule 1 leads to a decrease in insulin clearance, exacerbating hyperinsulinemia. Thus the decrease in Arhgap39/Porf-2 could be a result of increased suppression by insulin in the prediabetic state, although later, as insulin resistance with hyperinsulinemia advances to frank diabetes, Arhgap39/Porf-2 levels can be anticipated to rise. Both insulin and IGF-1 deficiencies have been linked to neurodegenerative (Biessels et al., 2014; Ekblad et al., 2017) and cardiovascular (Higashi et al., 2013) disease. This, in conjunction with the above findings, raises the question of whether increased *Arhgap39/Porf-2* expression factors into the pathophysiology of diabetic complications more generally.

### Potential physiologic and pathophysiological roles of Arhgap39/Porf-2

Tables 5 and 6 summarize the potential physiologic and pathophysiological roles of *Arhgap39/Porf-2*, based on the findings to date. Its wide phylogenetic and tissue distribution, its complex physiologic regulation, and its pivotal role in basic cell functions support the expectation that there is much more to discover about this particular RhoGAP.

**Table 5.** Potential physiologic roles of Arhgap39/Porf-2

Sexual dimorphism of hypothalamus
Axon guidance and directional migration
Regulation of early sperm development
Endothelial cell migration
Regulation of cell cycle in neural stem cells
Development of normal dendritic spine morphology
Cognitive performance
Development and function of extraembryonic structures

**Table 6.** Potential pathophysiological roles of Arhgap39/Porf-2

Congenital cognitive insufficiency
Age-related cognitive decline/neurodegeneration
Diabetic nephropathy
Diabetes-related cognitive dysfunction
Obesity-related hepatic dysfunction
Cognitive decline related to IGF-1 deficiency
Oncogenesis

## Summary

Arhgap39/Porf-2 (Vilse, CrossGAP) is a Rho GTPase activating protein that interacts with Rac1 and Cdc42. As is the case with many RhoGAPs, it has multiple functions. Among these are inhibition of cell proliferation, promotion of apoptosis, regulation of axon guidance and endothelial cell migration, and development of normal dendritic spine morphology in the hippocampus, which may underlie support of normal learning and memory. Various physiologic and hormonal cues direct region-specific expression in the brain, suggesting that day-to-day expression is carefully monitored and maintained. Finally, the finding that deletion of *Arhgap39/Porf-2* is fatal and the fact that this gene has been conserved through evolution for millennia highlight the crucial importance of this gene.

## Synthesis

Reviewing Editor: Margaret McCarthy, Univ of Maryland School of Medicine Decisions are customarily a result of the Reviewing Editor and the peer reviewers coming together and discussing their recommendations until a consensus is reached. When revisions are invited, a fact-based synthesis statement explaining their decision and outlining what is needed to prepare a revision will be listed below. The following reviewer(s) agreed to reveal their identity: Joshua Corbin, Nathalie Lamarche-Vane.

The authors present a comprehensive review of Arhgap39/Porf2/Vilse, a member of the Rho GTPase family, in a variety of biological functions. The review puts into perspective known information of this gene in a manner that is interesting and original. The review overall is well written and will provide a nice addition to the literature. Two reviewers provide a combination of suggestions for additional material and interpretations, as well as a number of specific editorial changes.

Overall comments/suggestions:

1) Both reviewers felt that Figure 1 is not all that informative. They suggested it be replaced with any of the following: a figure of Rac/Rho pathway and how Arhgap39 fits in this pathway, a figure of protein structure w/functional domains (referenced to Lines 146-211) and/or a figure with the structure of the protein modules and include the possible alternative splice variants.

2) The review focused on many aspects of Arhgap39/Porf2/Vilse, both neuronal and non-neuronal, thus the title maybe too specific.

3) Authors have not mentioned the tentative role of Arhgap39 in oncogenic processes (though this maybe getting too outside the theme of eNeuro). Arhgap39 is a gene involved in apoptosis and proliferation, related with the well-known tumor suppressor P53 and BAX, and several mutations has been reported in the Cosmic database associated to carcinogenesis. This information might be integrated in the text, as well as in table 6. Some of this information can be found at, for examples:

- http://cancer.sanger.ac.uk/cosmic/gene/analysis?ln=ARHGAP39

- IPA reports (Qiagen)

http://useast.ensembl.org/Homo_sapiens/Gene/Variation_Gene/Table?db=core;g=ENSG00000147799;r=8:144529179-144605816

- http://research-repository.uwa.edu.au/files/21715375/THESIS_DOCTOR_OF_PHILOSOPHY_JONES_Rachel_Maree_2017.pdf

4) Authors may also want to mention a role in morphogenesis (observed in Lee et al., 2017) and to add 'Morphogenesis' also in table 5.

5) It may also be worth mentioning the following study on Parkinson's disease https://link.springer.com/content/pdf/10.1007%2Fs10048-013-0383-8.pdf

6) As a general criticism, throughout the text it is difficult to follow to what species some of the assessments refer. E.g. lines: 300, 337, 354, 362, 376 (expression of what -protein/gene- in which species?), 389... Please, clarify.

7) Another general criticism along the text, to better clarify whether expression refers to gene or protein expression, i.e. using italics for gene expression of Arhgap39 (e.g. line 12).

8) Title in section V and table 4 'Upstream regulators' may be modified by something like 'Variables modifying Arhgap39 expression and upstream regulators'. Since 'Age' or 'sex' can be seen not as upstream regulators, but more as variables or conditions affecting Arhgap39 expression.

MiRs should be mentioned in this section as proper upstream regulators.

For more information on miR targeting Arhgap39, please refer to:

- IPA reports (Qiagen). Information at the end of this page.

- miR databases e.g. https://ccb-web.cs.uni-saarland.de/mirtargetlink/network.php?type=Target_Gene&qval=ARHGAP39

Specific line by line comments:

Lines 36, 438: In the text 'millennia' is mentioned as synonym of preservation through evolution. However, most of the genes, even if not preserved through evolution, have been preserved for millennia. E.g. line 11 could be modified by : 'Homologous or orthologous genes arehave been found in over 280 vertebrate and invertebrate species, suggesting preservation along evolution'. E.g. line 36: It Homologous or orthologous genes have been conserved for millennia along evolution with a wide phylogenetic distribution from invertebrate to mammals.

Line 45: phrase unclear. Maybe more clear including a sentence similar to Lee et al., 2017: "Rho GTPases switch between an active GTP-bound form and an inactive GDP-bound state". They in turn are modified by This switch is mediated by GTPase activating proteins (GAPs) and....

Line 54: Arabaidopsis

Line 63: Over the next 15 years (from Novak 1990 to 2014= 24 years). Or to date?

line 69-70: the statement "hypothalamic expression during a critical period of prenatal development is sex-specific" should include more details (ei what stages, what regions, specific differences between sexes, etc)

Line 80: Birds or fishes are not species. An Arhgap39/Porf-2-like gene was present in nine widely ranging animal species of vertebrates? including primates, ungulates, rodents, birds and fishes.

lines 78-81: it is stated that Arhgap39 was present in 9 species, but on line 111 it is stated that there are 180 orthologs in a variety of species. The numbers need clarification as there appears to be a disconnect.

Line 83: suggested to include synomym 'Rho GTPase activating protein 39'.

Line 175 (example): Throughout the text some sentences lack references.

Line 230: Since 2014 is now a while ago, suggested to remove "quite"

Line 232: "sightings" is not the most appropriate word in this context perhaps change to "gene expression studies"

Line 237-238: Please be more specific about "certain ganglia"

Line 255: These findings studies.

Line 281: BAX and P53: suggested to mention their role in protection against cancer progression.

Line 295: unclear paragraph.

Line 300: Lim aet al. (or Lim and Ritt).

Line 313: It maybe worth to mention what kind of alterations

Line 339: The subheading regarding age is a bit confusing. Is this the changes in levels of expression over developmental and post natal time? What does the RNA levels represent-total tissue? Examined by which type of analysis (qPCR?)

Line 353: suggested to add "differences" after sex

Line 437: since the gene deletion is constitutive, suggested to remove "developmental"

Table 2: Lines on column 1 on Peripheral Nervous system and Endocrine Organs not aligned with corresponding column 2.

Table 3: If possible it would be informative to add citations next to each of the functions so the reader can easily refer back to the original work.

Last page in pdf, is another figure? It appears cut off.

Page 1, line 7: a member of the Rho GTPase-activating protein group

Page 2, line 45: replace "modified" by regulated

Page 3, line 46: remove "that direct the binding and dissociation of GTP and GDP with the GTPases": this is not accurate.

Page 3, line 48: remove "GAPs promote the binding of GDP": this is wrong. GAPs enhance the intrinsic GTPase activity, leading to GTP hydrolysis and the inactive state of the GTPase.

Page 3, line 51: There are five small GTPase subfamilies, not superfamilies.

Page 3, line 53: replace superfamilies by subfamilies.

Page 3,line 49: replace GDP by guanine nucleotide

Page 12, line 250: replace modification by regulation

Page 12, line 252: replace "an activator of" by a negative regulator of
